# *In situ* molecular profiles of glomerular cells by integrated imaging mass spectrometry and multiplexed immunofluorescence microscopy

**DOI:** 10.1016/j.kint.2024.11.008

**Published:** 2024-11-20

**Authors:** Allison B. Esselman, Felipe A. Moser, Léonore E.M. Tideman, Lukasz G. Migas, Katerina V. Djambazova, Madeline E. Colley, Ellie L. Pingry, Nathan Heath Patterson, Melissa A. Farrow, Haichun Yang, Agnes B. Fogo, Mark de Caestecker, Raf Van de Plas, Jeffrey M. Spraggins

**Affiliations:** 1 Mass Spectrometry Research Center, Vanderbilt University, Nashville, Tennessee, USA; 2 Department of Chemistry, Vanderbilt University, Nashville, Tennessee, USA; 3 Delft Center for Systems and Control, Delft University of Technology, Delft, the Netherlands; 4 Department of Cell and Developmental Biology, Vanderbilt University, Nashville, Tennessee, USA; 5 Department of Biochemistry, Vanderbilt University, Nashville, Tennessee, USA; 6 Department of Pathology, Microbiology, and Immunology, Vanderbilt University Medical Center, Nashville, Tennessee, USA; 7 Department of Pediatrics, Vanderbilt University Medical Center, Nashville, Tennessee, USA; 8 Division of Nephrology and Hypertension, Department of Medicine, Vanderbilt University Medical Center, Nashville, Tennessee, USA; 9 Current affiliation: Aspect Analytics, C-Mine 12, 3600 Genk, Belgium.

**Keywords:** cellular analysis, glomeruli, immunofluorescence, lipidomics, MALDI IMS, multimodal imaging

## Abstract

Glomeruli filter blood through the coordination of podocytes, mesangial cells, fenestrated endothelial cells, and the glomerular basement membrane. Cellular changes, such as podocyte loss, are associated with pathologies like diabetic kidney disease. However, little is known regarding the *in situ* molecular profiles of specific cell types and how these profiles change with disease. Matrix-assisted laser desorption/ionization imaging mass spectrometry (MALDI IMS) is well-suited for untargeted tissue mapping of a wide range of molecular classes. Importantly, additional imaging modalities can be integrated with MALDI IMS to associate these biomolecular distributions to specific cell types. Here, we integrated workflow combining MALDI IMS and multiplexed immunofluorescence (MxIF) microscopy. High spatial resolution MALDI IMS (5 μm) was used to determine lipid distributions within human glomeruli from a normal portion of fresh-frozen kidney cancer nephrectomy tissue revealing intra-glomerular lipid heterogeneity. Mass spectrometric data were linked to specific glomerular cell types and substructures through new methods that enable MxIF microscopy to be performed on the same tissue section following MALDI IMS, without sacrificing signal quality from either modality. Machine learning approaches were combined enabling cell type segmentation and identification based on MxIF data. This was followed by mining of cell type or cluster-associated MALDI IMS signatures using classification and interpretable machine learning. This allowed automated discovery of spatially specific molecular markers for glomerular cell types and substructures as well as lipids correlated to deep and superficial glomeruli. Overall, our work establishes a toolbox for probing molecular signatures of glomerular cell types and substructures within tissue microenvironments providing a framework applicable to other kidney tissue features and organ systems.

Kidney physiology is driven by highly organized multicellular functional tissue units (FTUs) comprising the nephron. Probing the molecular profiles of specific tissue features across spatial scales, from entire FTUs to cell types, while maintaining spatial context, is critical for understanding both normal and diseased cellular functions. For example, glomeruli are complex FTUs that filter blood with the help of unique cell types, such as podocytes, mesangial cells, and fenestrated endothelial cells, organized around the glomerular basement membrane.^1^ Diseases of the kidney, such as diabetic kidney disease, can alter glomeruli, leading to podocyte loss, expansion of the mesangium, and thickening of the glomerular basement membrane.^2-4^ Determining the distribution of biomolecules among these cell types in healthy glomeruli is necessary to better understand cellular changes in diseased states.^5^ Imaging technologies are emerging to address the challenge of defining molecular characteristics of tissue features with increasing specificity.^6^ Several large-scale consortia, including the Human Biomolecular Atlas Program^7^ and the Kidney Precision Medicine Project,^8^ are applying these approaches to construct molecular atlases of the human kidney.

Immunofluorescence microscopy is commonly used to map the distribution of specific proteins and delineate the cellular organization of tissues.^9^ Recent advancements in highly multiplexed methods allow more comprehensive spatial cell typing and the ability to reveal cellular organization. However, multiplexed immunofluorescence (MxIF) is targeted and limited to proteins, omitting other key molecular classes.^10^ Matrix-assisted laser desorption/ionization imaging mass spectrometry (MALDI IMS) addresses this limitation by enabling untargeted, high spatial resolution imaging (<10 μm pixel sizes) of drugs, metabolites, lipids, glycans, and proteins, making it ideally suited for molecular discovery.^11,12^ Multimodal approaches combining MALDI IMS and MxIF allow hundreds of molecular features detected by IMS to be associated with specific tissue features and cell types.

MALDI IMS and MxIF are traditionally performed on serial sections. As modern instrumentation enables higher spatial resolution IMS, there is a greater need for multimodal imaging experiments to be performed on the same tissue section. Although serial tissue sections remain appropriate for comparing larger-scale tissue features, cellular structures at a given location can change substantially between serial sections. Initial examples of these workflows have shown the potential for IMS-MxIF–integrated workflows but were limited in their spatial specificity and strategies for cross-modality data mining.^13-15^ Here, we demonstrate integrated methods for performing high spatial resolution MALDI IMS and MxIF on a single tissue section, allowing IMS-reported molecular distributions to be directly correlated with MxIF-delineated tissue features, enabling automated discovery of *in situ* molecular marker candidates for glomerular cell types and substructures.

## METHODS

Tissue sections were collected from a normal portion of fresh-frozen renal cancer nephrectomy tissue and thaw-mounted onto glass slides. The sections were prepared for positive ion mode MALDI IMS, sampling only glomeruli as previously described.^16^ Immediately after IMS, sections were fixed for batch MxIF (cyclic-IF)^17^ analysis using 10 antibodies across 3 cycles. The immunofluorescence intensity signatures were clustered, segmenting the glomeruli into substructures, including specific glomerular cell types. Subsequently, a classification model was trained to recognize MxIF-based segments using IMS measurements as inputs. The model was interpreted using Shapley additive explanations (SHAP),^18,19^ and a global SHAP score was calculated for each of the IMS-measured molecular features, generating a ranked list of relevant biomarker candidates for each glomerular segment (i.e., cell type and substructure). See Supplementary Methods and Supplementary Tables S1 and S2 for details.

## RESULTS

**Figure 1 ∣:**
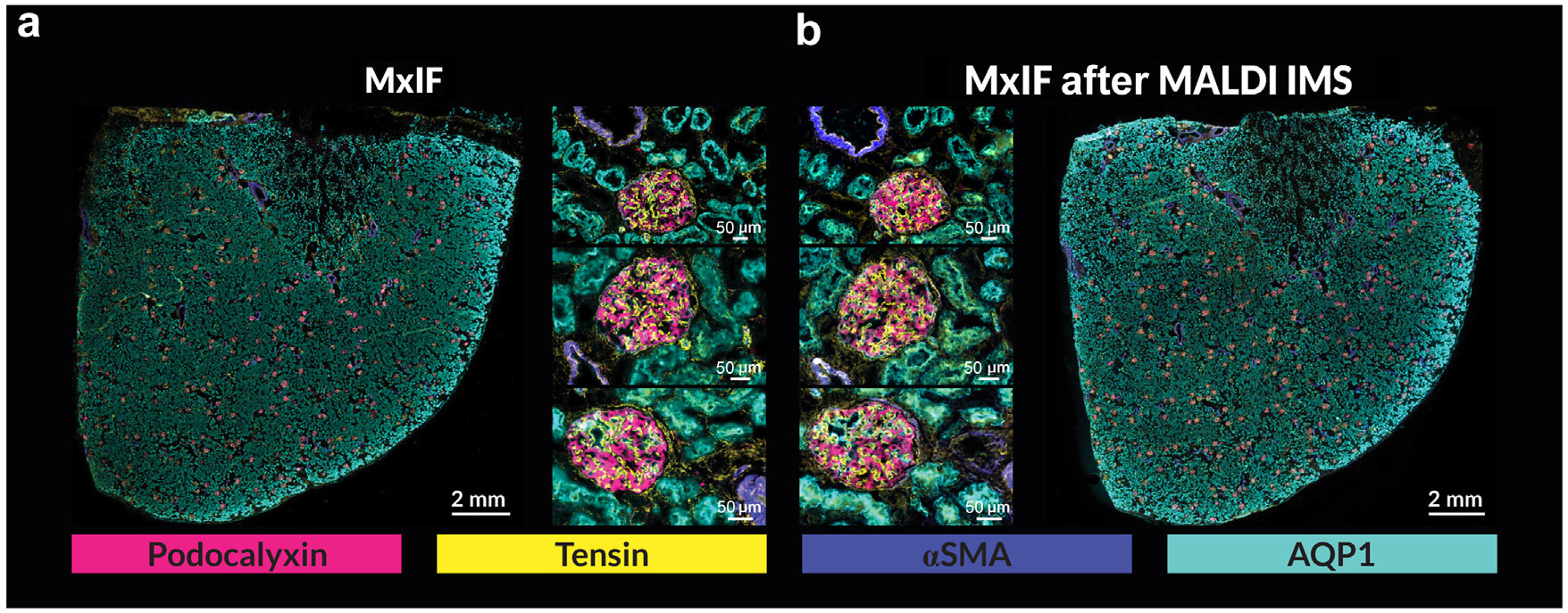
MxIF quality after MALDI imaging mass spectrometry. Comparison of serial human kidney tissue sections, one only stained and imaged using MxIF (**a**) and the other imaged after MALDI IMS measurement of the glomeruli and then stained and imaged using MxIF (**b**). Four of the 10 antibodies are represented in the highlighted images. Selected regions from the whole slide images that include individual glomeruli (serial images of the same glomeruli) are provided to compare the stain quality between the 2 experiments. The stain quality of the 4 represented antibodies was minimally impacted by the MALDI IMS experiment and its accompanying sample preparation. The 6 μm difference associated with the sectioning thickness between the serial sections shows differing patterns of podocalyxin (podocytes, pink) and tensin (mesangial cells, yellow) between the 2 serial sections, emphasizing the importance of performing multimodal imaging on the same tissue section. αSMA, α-smooth muscle actin; AQP1, Aquaporin 1, a protein coding gene; MALDI IMS, matrix-assisted laser desorption/ionization imaging mass spectrometry; MxIF, multiplexed immunofluorescence. To optimize viewing of this image, please see the online version of this article at www.kidney-international.org.

High spatial resolution IMS methods were optimized to minimize tissue damage from laser irradiation. After IMS acquisition, MxIF was performed to map specific cell types and structures within and surrounding the glomeruli (Table 1 and Supplementary Table S3). Figure 1a and b shows MxIF data from serial sections without and with a preceding IMS measurement. The comparison demonstrates the retention of MxIF stain quality after MALDI IMS for the selected antibodies. IMS data were acquired first as the MxIF workflow can alter the molecular milieu of the tissue, reducing the capacity to perform subsequent spatial omics experiments.

Autofluorescence images were used to automatically outline glomerular tissue areas for MALDI IMS measurement as previously described.^16,20^ This enabled rapid acquisition of intraglomerular lipid distributions for >250 glomeruli per tissue section.^16,20^ After IMS, tissue sections were stained with the antibody panel and imaged. Supplementary Figure S1 shows the whole-slide autofluorescence image, glomerular measurement regions, MALDI IMS, and MxIF of a single tissue section.

**Figure 2 ∣:**
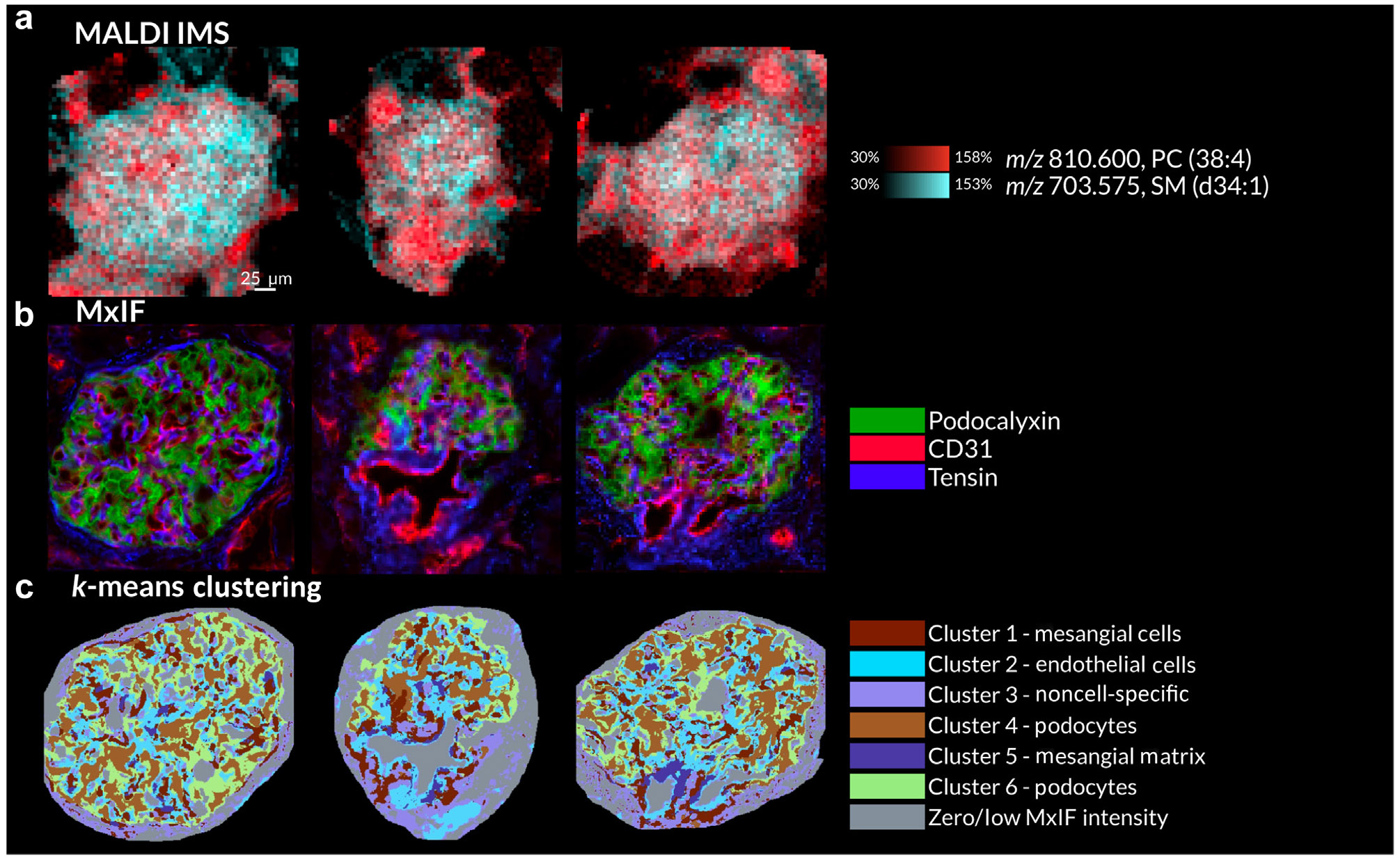
Multimodal molecular imaging data and segmentation maps from 3 selected glomeruli. MALDI ion images for PC(38:4) (*m/z* 810.600) and SM(d34:1) (*m/z* 703.575) highlight intraglomerular molecular heterogeneity (**a**). Overlaid MxIF images of podocalyxin, CD31, and tensin (**b**). All *k*-means clustering-based segments of the MxIF data and, where possible, the glomerular cell type that dominates each segment (**c**). MALDI IMS, matrix-assisted laser desorption/ionization imaging mass spectrometry; MxIF, multiplexed immunofluorescence. To optimize viewing of this image, please see the online version of this article at www.kidney-international.org.

**Figure 3 ∣:**
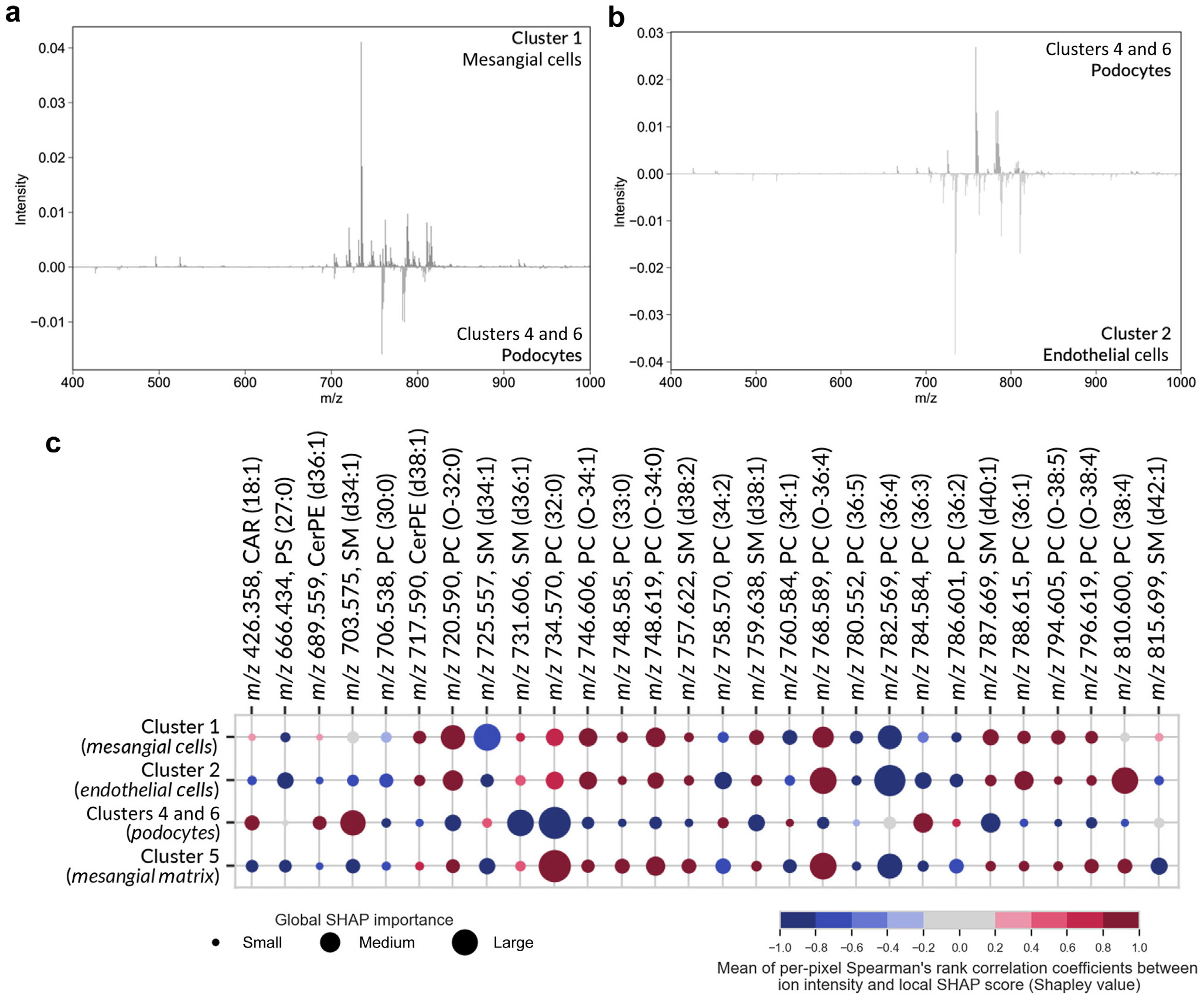
Molecular profiles specific to glomerular segments are revealed by integrating IMS and MxIF data. The mass spectral difference plots show the average ion intensity differences between cluster 1 (primarily mesangial cells) and combined clusters 4 and 6 (primarily podocytes) (**a**), and clusters 4 and 6 (primarily podocytes) versus cluster 2 (primarily endothelial cells) (**b**). The bubble plot from the SHAP analysis (**c**) summarizes the biomarker candidates for the glomerular segmentations and their dominant cell types and substructures. The size of each bubble indicates the global SHAP importance of a given ion species (column) to recognizing a given glomerular subarea (and its dominant cell type) (row), and the color indicates a positive (red) or negative (blue) correlation of the ion species abundance to that cluster’s recognition. This analysis shows that every glomerular segmentation has its own unique profile of relevant IMS-measured molecular species. IMS, imaging mass spectrometry; MxIF, multiplexed immunofluorescence; SHAP, Shapley additive explanations.

MxIF pixels within glomerular measurement regions were clustered using *k*-means clustering based on the fluorescence intensities of tensin, podocalyxin, fibronectin, CD31, synaptopodin, and nestin, delineating 6 subglomerular segments, some of which were enriched for specific glomerular cell types. Where possible, a segment’s standardized mean antibody fluorescence intensity profile (Supplementary Figure S2) was used to link it to a corresponding cell type. Three glomeruli are highlighted in Figure 2, displaying lipid distributions uncovered by MALDI IMS (Figure 2a), protein distributions from MxIF (Figure 2b), and glomerular segments based on clustering of the MxIF data (Figure 2c). Data from all modalities were spatially coregistered, allowing the extraction of MALDI IMS pixels specific to each MxIF-based glomerular segment and the generation of an average mass spectrum for each associated substructure and cell type. The average differences in ion intensity between cluster segments (i.e., cell type or substructure) are shown in Figure 3a-b and Supplementary Figures S3-S8.

To discover multivariate molecular profiles distinctive for glomerular cell types and substructures, MALDI IMS and MxIF data were integrated using interpretable supervised machine learning. The MxIF-based segments were used as labels for each IMS pixel, and a classification model was trained to differentiate glomerular segments (and their dominant cell types) based on IMS-reported molecular ions. The mean balanced accuracy, F1-score, precision, and recall were >85% for all segments (see Supplementary Table S4 for classification model performance metrics). Subsequently, SHAP was used to interpret the model and discover biomarker candidates for each glomerular cluster.^18^ Given the model, SHAP ascertains the degree (relevance) and the direction (positive or negative correlation) of influence every ion has on the recognition of a particular glomerular segment.^18^ A global SHAP score was calculated for each IMS-reported molecule, quantifying its relevance to recognizing a certain segment and providing a ranked list of biomarker candidates for each glomerular cell type or substructure (see Supplementary Figures S9-S12). These data can be represented as a bubble plot (Figure 3c), where the size of each bubble indicates the global SHAP importance of a given molecule (column) for recognizing a given glomerular segment (row). The color indicates a positive (red) or negative (blue) correlation of the molecule’s abundance to the recognition of that segment. Supplementary Figure S13 shows the entire SHAP bubble plot, and Supplementary Table S5 summarizes the identification details for each molecule represented in the SHAP outputs.

## DISCUSSION

We demonstrate an advanced multimodal workflow combining MALDI IMS, MxIF, and interpretable machine learning to uncover *in situ* molecular profiles of specific cell types and substructures of FTUs, here aimed at intra-glomerular features. Our methods maintain proper antibody staining after MALDI IMS analysis, allowing multiple imaging modalities to sample the same tissue features (not assured when using serial sections) and promoting conservative use of precious tissue samples. Multivariate SHAP analysis provides a unique fingerprint of positively and negatively correlated molecular species for every segmented tissue feature (Figure 3c). Interpreting the data in this way allows high-dimensional spatial omics data to be mined efficiently and potential biomarker candidates to be readily discerned for each substructure or cell type. For instance, phosphatidylcholine PC(38:4) detected at *m/z* 810.600 was determined to be a positively correlated biomarker candidate for the endothelial cell–related segment and a negatively correlated marker for the podocyte-related segment. In contrast, sphingolipid SM(d34:1) (*m/z* 703.575) was a positively correlated biomarker candidate for the podocyte-related segment. Sphingolipids play a role in podocyte homeostasis, mediating normal and disease-related responses.^21^ The ceramide chain of SM(d34:1) is synthesized by ceramide synthase 6 (CERS6), the expression of which is critical for podocyte cytoskeletal organization and maintenance of slit diaphragms.^22^ Based on our observations of SM(d34:1) as a robust glomerular biomarker candidate and the previously established molecular relationship of CERS6 with podocytes, we hypothesize that SM(d34:1) is a critical lipid regulator of podocyte structure and function. This method also reveals molecular differences associated with spatial location and localized disease. To demonstrate this, we performed an intrasample comparison of 30 deep and 30 superficial glomeruli. A binary classification model for each cell segment was built and interpreted with SHAP to assess cell type–specific lipid differences among these 2 groups. Bar plots showing the molecular markers correlated with deep and superficial glomerular cell types and SHAP performance metrics can be found in Supplementary Figures S14-S17 and Supplementary Table S6. This analysis uncovered a positive correlation of multiple fatty acylcarnitine species with cell types of deep glomeruli. Because carnitine is known to be a key driver of metabolic activity in cells,^23^ this observation could lead to studies investigating the connection between these carnitine species, the metabolic state of long-looped nephrons, and the functional activity of long-looped nephrons associated with the medullary vasa recta.^24^ The ability to unveil these connections between biomarker candidates and known biology points to the potential for our integrated approach to serve as a molecular discovery tool for advancing the understanding of mechanisms of cellular function within tissue microenvironments.

As the most complex kidney FTU, glomeruli serve as a challenging case study demonstrating the broad applicability of our workflow. It can be readily adapted to analyze other FTUs, including tubules and ducts in the kidney, as well as other organ types. Although this proof-of-concept study was performed on healthy tissue, it could also be applied to studying diseases such as chronic kidney disease. For example, as diabetic kidney disease progresses, glomeruli undergo podocyte loss and mesangial cell expansion, but the cellular-level lipidomic changes that occur are not well characterized.^25^ Our workflow can be used to find specific biomarker candidates for renal cell types in a spatial context. This could be used to subtype diabetic kidney disease and other kidney diseases. In essence, our toolbox offers insight into the complex relationship between the molecular and cellular organization of tissues, paving the way for precision medicine by uncovering how these relationships are altered in normal aging and disease.

## Supplementary Material

Supplemental Material

Supplementary material is available online at www.kidney-international.org.

## Figures and Tables

**Table 1 ∣:** Antibody panel for MxIF microscopy

Target	Cell types/structures	Cycle	Fluorophore	Conjugation type
Collagen IV α1/2	Tubular basement membrane, mesangial matrix, and glomerular capsule	1	Cy5	Indirect
Collagen IV α5	Glomerular basement membrane, glomerular capsule, and collecting duct and distal convoluted tubule basement membrane	1	Cy3	Indirect
Tensin	Mesangial cells and vascular smooth muscle cells	1	AF 488	Indirect
Podocalyxin	Podocyte cytoplasm and plasma membrane	2	AF 488	Direct
Fibronectin	Mesangial matrix and muscularized vessel walls	2	AF 594	Direct
CD31	Endothelial cells	2	AF 647	Direct
Synaptopodin	Podocyte cytoplasm and plasma membrane	2	Cy7	Direct
Nestin	Podocyte cytoplasm	3	AF 488	Direct
αSMA	Vascular smooth muscle cells	3	AF 594	Direct
AQP1	Proximal tubules	3	AF 647	Direct

αSMA, α-smooth muscle actin; AQP1, Aquaporin 1, a protein coding gene; MxIF, multiplexed immunofluorescence.

## Data Availability

The data are available at the National Institutes of Health Human Biomolecular Atlas Program (HuBMAP) data portal (HuBMAP portal: doi:10.35079/HBM793.WWZC.833, doi:10.35079/HMB958.SHCP.655, doi:10.35079/HBM697.PXDS.833, doi:10.35079/HBM524.VXVH.562; MxIF: https://doi.org/10.48539/HBM624.CHLG.652, https://zenodo.org/records/11245254).

## Translational Statement

Here, we demonstrate an integrated multimodal molecular imaging workflow combining untargeted imaging mass spectrometry and multiplexed immunofluorescence microscopy onto a single tissue section to enable the discovery of lipid profiles associated with glomerular cell types and structures. This method establishes a toolbox for precision medicine, allowing for the discovery of cell type–specific molecular profiles within tissue microenvironments. This framework can be applied to study the localization and alterations of biomolecules among cell types of the kidney, and the resulting spatially derived lipid biomarker candidates elucidate the relationship between renal cellular organization and molecular distributions.
