# Supplementary material for: In situ molecular profiles of glomerular cells by integrated imaging mass spectrometry and multiplexed immunofluorescence microscopy
Source: Kidney Int. Author manuscript; Available in PMC 2026 Apr 13. (PMC13072436; doi:10.1016/j.kint.2024.11.008)
Supplement: Supplemental Material [file NIHMS2159606-supplement-Supplemental_Material.pdf]

Supporting Information

Revealing *In Situ* Molecular Profiles of Glomerular Cell Types and Substructures with Integrated Imaging Mass Spectrometry and Multiplexed Immunofluorescence Microscopy

Allison B. Esselman[1,2], Felipe A. Moser[3], Léonore E. M. Tideman[3], Lukasz G. Migas[1,3], Katerina V. Djambazova[1,4], Madeline E. Colley[1,5], Ellie L. Pingry[1,4], Nathan Heath Patterson[1,5,#], Melissa A. Farrow[1,4], Haichun Yang[6,7], Agnes B. Fogo[6,7,8], Mark de Caestecker[4,8], Raf Van de Plas[1,3,5*], Jeffrey M. Spraggins[1,2,4,5,6]*

[1] Mass Spectrometry Research Center, Vanderbilt University, Nashville, TN 37235, USA
[2] Department of Chemistry, Vanderbilt University, Nashville, TN 37235, USA
[3] Delft Center for Systems and Control, Delft University of Technology, 2628 CD Delft, The Netherlands
[4] Department of Cell and Developmental Biology, Vanderbilt University, Nashville, TN 37232, USA
[5] Department of Biochemistry, Vanderbilt University, Nashville, TN 37205, USA
[6] Department of Pathology, Microbiology, and Immunology, Vanderbilt University Medical Center, Nashville, TN 37205, USA
[7] Department of Pediatrics, Vanderbilt University Medical Center, Nashville, TN 37232, USA
[8] Division of Nephrology and Hypertension, Department of Medicine, Vanderbilt University Medical Center, Nashville, TN 37232, USA
[#]Current affiliation: Aspect Analytics, C-Mine 12, 3600 Genk, Belgium

*Jeffrey M. Spraggins; 465 21st Ave S. Room 9160, Medical Research Building III Vanderbilt University, Nashville, TN 37240; Fax: 615-343-8372; Phone: 615-343-9207; jeff.spraggins@vanderbilt.edu

*Raf Van de Plas; Delft University of Technology, Delft Center for Systems and Control, Mechanical Engineering Faculty, Mekelweg 2 – Gebouw 34, 2628 CD, Delft, The Netherlands; raf.vandeplas@tudelft.nl

Additional supplemental figures can be found at *https://doi.org/10.6084/m9.figshare.27016291.v1*

Table of Contents

Method S1	Extended Methods	S-3 – S-7
Table S1	qTOF Instrument Parameters	S-7
Table S2	LC-MS/MS Negative and Positive Ion Mode Instrument Parameters	S-7
Table S3	Antibody Information	S-8
Figure S1	Whole Slide Imaging Workflow	S-8
Figure S2	Standardized Mean Fluorescent Intensity Bar Plot	S-9
Figure S3	Plot of Spectral Differences between Cluster 1 and 2	S-9
Figure S4	Plot of Spectral Differences between Cluster 1 and Clusters 4 and 6	S-10
Figure S5	Plot of Spectral Differences between Clusters 4 and 6 and Cluster 2	S-10
Figure S6	Plot of Spectral Differences between Cluster 5 and 1	S-11
Figure S7	Plot of Spectral Differences between Cluster 5 and 2	S-11

Figure S8	Plot of Spectral Differences between Cluster 5 and Clusters 4 and 6	S-12
Table S4	SHAP Performance Metrics	S-12
Figure S9	Ranked SHAP Values for Cluster 1	S-13
Figure S10	Ranked SHAP Values for Cluster 2	S-13
Figure S11	Ranked SHAP Values for Clusters 4 and 6	S-13
Figure S12	Ranked SHAP Values for Cluster 5	S-14
Figure S13	Full, Unlabeled SHAP Bubble Plot	S-14
Table S5	Lipid Identification Table	S-15
Figure S14	Ranked SHAP Values for Short versus Long Looped Cluster 1	S-16
Figure S15	Ranked SHAP Values for Short versus Long Looped Cluster 2	S-16
Figure S16	Ranked SHAP Values for Short versus Long Looped Clusters 4 & 6	S-16
Figure S17	Ranked SHAP Values for Short versus Long Looped Cluster 5	S-17
Table S6	Binary Classification SHAP Performance Metrics	S-17

Method S1

Materials. Ammonium formate, carboxymethylcellulose sodium (CMC), glycerol, 30% hydrogen peroxide, sodium hydroxide, 10% neutral buffered formalin (NBF), and a periodic acid-Schiff (PAS) stain kit was purchased from Sigma-Aldrich (St. Louis, MO). The matrix 4-(dimethylamino)cinnamic acid (DMACA) with 99% purity and 20mM Hoechst 3342 were bought from Thermo Scientific (Waltham, MA). High-performance liquid chromatography (HPLC)-grade acetone, isopentane, and Superfrost Plus microscope slides were purchased from Fisher Scientific (Pittsburg, PA). Universal blocking reagent (10x) was purchased from BioGenex Laboratories (Fremont, CA), and antibody diluent reagent solution was purchased from Invitrogen (Waltham, MA). Secondary antibodies: anti-rat conjugated with Cy3, anti-mouse conjugated with Cy5, and anti-rabbit conjugated with AF 488 were purchased from Jackson Immuno Research (West Grove, PA). A Lightning-Link antibody conjugation kit for Cy7 was purchased from Abcam (Cambridge, UK). Equisplash (330731), cardiolipin (791108C), and sulfatide (860736) standards were purchased from Avanti Polar Lipids (Birmingham, AL). Water, methanol, acetonitrile, 2-propanol, methyl tert-butyl ether (MTBE), and ammonium formate (for LC-MS/MS) were purchased from EMD Millipore (Burlington, MA); formic acid (for LC-MS/MS) was purchased from Thermo Scientific (Rockford, IL). Human kidney samples were provided by the Cooperative Human Tissue Network at Vanderbilt University Medical Center. **Table S3** provides additional antibody information.

Sample and Data Collection. De-identified human kidney samples were obtained from a disease-free tumor associated nephrectomy from a 57-year-old white male with a BMI of 25.3. Samples were collected by the Vanderbilt Cooperative Human Tissue Network (CHTN, IRB Protocols #181822 "Biomolecular Multimodal Imaging for 3-Dimensional Tissue Mapping of the Human Kidney") and placed on ice within 1-2 hours of surgery. Tissue blocks (~0.5cm × 1cm × 2-4cm) were cut extending from the cortex through the medulla on their long axis, and frozen in 2.6% CMC in water on a dry ice/isopentane slurry and stored at -80C prior to sectioning.[1] Serial 6 µm thick cryosections were cut using a CM3050 S cryostat (Leica Biosystems, Wetzlar, Germany) and thaw-mounted onto Superfrost Plus microscope slides. Autofluorescence images were acquired of each section using standard DAPI, eGFP, and DSRed fluorescent filters on a Zeiss AxioScan.Z1 slide scanner (Carl Zeiss Microscopy GmbH, Oberkochen, Germany), equipped with a Colibri7 LED light source.

MALDI IMS Preparation. Immediately after the autofluorescence images were collected three serial sections were prepared for MALDI IMS analysis. The sections were submerged in chilled (4 °C) 150 mM ammonium formate 3 times for 45 seconds each, then dried with nitrogen gas. An in-house developed sublimation device was used to sublime 5 mg of 4-(dimethylamino)cinnamic acid (DMACA) onto the slide. The apparatus was heated (190 °C) for 10 minutes under vacuum (110-150 mTorr) while the sample was cooled to -78 °C using a dry ice and acetone slurry in the cold finger, resulting in a final matrix density of ~0.22 µg/mm^2.[2] Functional tissue unit (FTU) segmentation of glomeruli was performed using the autofluorescence image of each tissue section using a previously described method.[3–5] The FTU segmentations were used to define measurement regions in flexImaging so that only the glomeruli were measured by MALDI IMS. The FTU segmented areas were scaled by a factor 1.6 using an affine scale transform to allow for slight errors in spatial targeting and to ensure capture of the border of each glomerulus. The MALDI IMS experiments were performed on a timsTOF fleX mass spectrometer equipped with a microGRID stage (Bruker Daltonik, Bremen, Germany).[6] Tissue imaging data were collected at 5 µm × 5 µm pixel size with no beam scan, a 5 µm pitch, 25 shots per pixel, and 4.8% total laser

power. Lipid data were collected in positive ion mode from *m/z* 400 to 2000. Instrument-specific parameters are available in **Table S1**. Molecular annotations were supported by LC-MS/MS. Selected IMS peaks were linked to LC-MS/MS data using mass accuracy (<5 ppm). After MALDI IMS data acquisition, post-IMS autofluorescence images were acquired on the same tissue sections prior to matrix removal using a Zeiss AxioScan.Z1 fluorescence slide scanner using the previously described eGFP fluorescence filter and a monochromatic brightfield image. After the collection of the post-IMS autofluorescence images, all tissue sections underwent the MxIF protocol.

LC-MS/MS. To enhance the rigor of the lipid identifications and because of the inability to identify isomeric and some isobaric species with MALDI IMS, liquid chromatography with tandem mass spectrometry (LC-MS/MS) was performed. Two fresh frozen 10 μm thick tissue sections were added to glass vials along with 5 μL of 100 μg/mL internal standards Avanti Equisplash, C24:1 mono-sulfo galactosyl(ß) ceramide-d7 (d18:1/24:1) (Avanti), and 18:2 Cardiolipin-d5 (Avanti) and methanol was added until the tissue was submerged. Metal beads were added to the vials and the tissue was homogenized by vortexing for 1 minute, placing the vials on dry ice for 5 minutes, and then sonicating on ice for 30 minutes. Lipids were extracted by adding 1600 μL of ice cold MTBE and 400 μL of ice cold water to 400 μL of ice cold methanol. Vials were centrifuged at 100 × *g* for 10 minutes at 4 °C and then allowed to rest on ice for 10 minutes. The top layer (1400 μL) was extracted to a new vial and dried under nitrogen. The sample was resuspended in 300 μL of methanol. Reversed-phase separation was performed on a Waters Premier UHPLC with a 2.1×100 mm Waters Premier CSH-C18 column heated to 60 °C. Mobile phase A consisted of 60:40 ACN:H_2O with 10 mM ammonium formate and 0.1% formic acid. Mobile Phase B consisted of 90:10 IPA:ACN with 10 mM ammonium formate and 0.1% formic acid. The total analysis time was 20 minutes, followed by a 10 minute equilibration time. A timsTOF fleX mass spectrometer utilized parallel accumulation-serial fragmentation (PASEF) and MS/MS stepping to obtain the collision cross section (CCS) of each species to permit the simultaneous measurement of both low *m/z* fragments and higher molecular weight lipids. Instrument-specific parameters can be found in **Table S2.** Analysis was performed with MS-DIAL version 4.80 and all lipid annotations were blank filtered, retention scored, and referenced matched with the MS-DIAL combined database.

Cyclic MxIF and Histological Stain Preparation. Cyclic MxIF was performed using a modified protocol.[7] Briefly, tissue sections were fixed with 10% NBF by submerging the tissue for 5 minutes. The tissue sections were then washed with 1X PBS, blocked for 30 minutes with 1X universal blocking reagent, and then incubated with the first cycle of antibodies overnight at 4 °C (see **Table S1** for MxIF cycle and antibody information). Tissue sections were then washed with 1X PBS and incubated with secondary antibodies for 60 minutes for any unconjugated primary antibodies. The sections were washed with 1X PBS and incubated with Hoechst for 10 minutes before attaching a coverslip using a 50:50 glycerol:H_2O solution. Each cycle was imaged using a Zeiss AxioScan.Z1 fluorescence slide scanner using standard AF488, AF594, AF647, Cy3, Cy5, Cy7, and DAPI filters. The coverslips were removed by soaking the slides in 1X PBS. The fluorophores were inactivated between each cycle by laying the slides over an LED light and pipetting a solution of basic 0.1 M sodium bicarbonate with 3% hydrogen peroxide in deionized water. After an hour, the slides were washed with 1X PBS and incubated overnight at 4 °C with the next cycle of antibodies. This process continued until 3 cycles had been imaged. Histograms from each image were adjusted individually as best fit for **Figures S1-S11**. After the last cycle was imaged, the coverslip was removed by submerging the slide in 1X PBS, and the tissue section was stained with PAS.[8]

Multimodal Image Registration and Data Analysis. MALDI IMS and microscopy images were registered in a two-step process. First, MALDI IMS pixels were aligned to laser ablation marks, as measured by the post-IMS autofluorescence image, using in-house developed software *image2image* to manually select corresponding pairs of laser ablation marks and IMS pixels. Additional microscopy images were then registered to the IMS data via the previously registered post-IMS autofluorescence images in the *wsireg* software.[9] All registered whole-slide images were stored in the vendor-neutral pyramidal OME-TIFF format and maintained their original spatial resolution (i.e., there is no downsampling or loss of pixel spacing through the registration process). After alignment of all images, previously used automated glomeruli segmentations, as predicted by a convolutional neural network, were used to find the MALDI IMS pixels associated with each detected glomerulus and were extracted for further data analysis.

***k*-Means Clustering of Glomeruli Pixels.** Glomerular substructures (and, where possible, their corresponding cell types) were segmented by clustering the fluorescent intensities of MxIF antibodies. First, glomeruli-specific antibody channels (tensin, podocalyxin, fibronectin, CD31, synaptopodin, and nestin) of each image were manually thresholded to remove the majority of the background and non-specific binding intensities. Then, the previously described glomeruli segmentations were used to mask the glomeruli of each image, resulting in a total of 67,269,568 pixels of N=6 dimensions (one for each of the channels above). The 27,491,379 pixels that were below the manual threshold across all channels were separated into their own cluster. The intensities of the remaining pixels were standardized on a per-channel basis before clustering them using a standard *k*-means algorithm. This was repeated for clusters ranging from k=2 to k=15, the optimal of which was determined to be k=6 by using the elbow method. The segmentation masks were then generated by placing an integer value between 1 and 6 in the location of each pixel in the original image, depending on which cluster they belong to. A value of 0 was given to the thresholded pixels that were excluded from the *k*-means clustering to distinguish them from the rest. Finally, each class of the segmentation masks was refined by performing a single iteration of binary dilation followed by a single iteration of binary erosion and removing objects that were smaller than 6 pixels.

MALDI IMS Data Preprocessing. MALDI IMS data were exported from the Bruker timsTOF file format (.d) to a custom binary format. Each pixel/frame contained centroid peaks that covered the entire acquisition range and was reconstructed to form a pseudo-profile mass spectrum using Bruker's SDK (v2.21). The data were *m/z* aligned using 6 peaks that appeared in at least 50% of the pixels using the *msalign* library (v0.2.0). The mass axis of the dataset was calibrated, using theoretical masses for the 6 peaks, to approximately ±1 ppm precision. The MALDI IMS data was subsequently normalized using a total ion current (TIC) approach. Then, an average mass spectrum based on all pixels belonging to each *k*-means cluster in the data set was computed. The average mass spectrum was peak-picked and a total of 1161 peaks were detected. Note that isotopic peaks were not removed in the classification workflow.

Supervised Machine Learning and Shapley Additive Explanations. Clusters 1, 2, 3, 5, and the combination of 4 and 6 were used as classes to build eXtreme Gradient Boosting (XGBoost) classification models that recognize one of the 5 clusters. The total MALDI IMS pixels for each cluster were 48,141 pixels in cluster 1, 100,292 pixels in cluster 2, 183,264 pixels in cluster 3, 9,786 pixels in cluster 5, and 306,936 pixels in the combination of clusters 4 and 6. We take the one-versus-all approach to multiclass classification: each classification task differentiates one cluster from the other four clusters (imbalanced binary classification). Our chosen interpretability method, Shapley additive explanations (SHAP), enables us to determine which ion species have a

marker-like relationship to each cluster by quantifying each ion species' importance to the corresponding classification models and the task of recognizing a certain MxIF-delivered glomerular cluster. For a given classification (i.e., cluster recognition) task, SHAP measures the global (experiment-wide) and local (per-pixel) relevance, or importance, of each ion species. The global SHAP importance scores (hereafter referred to as SHAP scores) are used to rank all IMS-provided ion species by decreasing relevance to recognize a specific cluster, and the top ions of the list can be used to select a subset of highly discriminative molecular species that represent potential biologically relevant marker candidates for the cluster in question. The local SHAP importance scores, or local Shapley scores, are used to measure the direction of relevance (by positive or negative monotonic correlation) and to assess the significance of the relationship between a given molecular species' ion intensity measurements and a pixel's likelihood of belonging to a given cluster.

The first step of the classification-and-interpretation workflow is cluster recognition. Rather than use one XGBoost model, we use an ensemble of 10 XGBoost models for each one-versus-all classification task. The training process of each of these XGBoost models is initialized using a different random seed, and the models are trained on slightly different training datasets. The ten training datasets differ in the following two ways: random under-sampling of the majority class (and, in case of extreme class imbalance, oversampling of the minority class by synthetic data augmentation) is done with ten different random seeds, and the split between training and testing datasets is done by sampling without replacement with ten different random seeds. Spurious patterns due to correlated inputs tend to cancel out across the different XGBoost models, whereas true correlations tend to be reinforced by the ensembling process. The patterns that dominate the decision-making process (and the ensuing SHAP explanations) of the ensemble are therefore more likely to be biologically relevant.

The second step of the workflow consists in quantifying the importance of each ion species with respect to a given classification task. We apply SHAP to the ensemble of ten XGBoost models obtained in the previous step. For each XGBoost model, SHAP returns one global SHAP importance score per molecular species: the global SHAP importance score is obtained by taking the mean of the magnitude of the local SHAP importance scores, or Shapley values, across all pixels making up the sample. The total global importance score of an ion species, which we previously referred to as the SHAP score, is the mean of the ten global SHAP scores obtained for that species from the ten XGBoost models making up the ensemble. The SHAP score, therefore, provides an experiment-wide measure of an ion species' predictive importance with respect to a given classification task.

We translate the task of biomarker candidate discovery into a feature selection problem: the molecular species are ranked in descending order of SHAP score to facilitate the selection of a shortlist of molecular species that may be useful biomarker candidates. In the summary bubble plots, the size of each marker corresponds to the global SHAP importance score of a given molecular species (column) for a given dataset (row). The next question is whether a biomarker candidate is positively or negatively correlated with a given target characteristic. We measure the direction and magnitude of the relationship by computing the Spearman rank-order correlation coefficient $\boldsymbol{\rho}$ between the mean-centered intensity and the Shapley values of a given molecular species. The Spearman rank-order correlation coefficient $\boldsymbol{\rho}$ also provides a way of assessing the statistical significance of the relationship: $\boldsymbol{\rho}$ ranges from -1 to 1, and we consider $\boldsymbol{\rho}$ to be significant if its magnitude exceeds 0.2. In the bar charts and summary bubble plots, the marker color

corresponds to the Spearman rank-order correlation coefficient per molecular species (column) and per dataset (row).

Table S1. Instrument Parameters for Positive Ion Mode qTOF MALDI IMS Experiments.

**Positive Ion Mode**	
Transfer	
MALDI Plate Offset	70.0 V
Deflection 1 Delta	70.0 V
Funnel 1 RF	400.0 Vpp
isCID Energy	5.0 eV Vpp
Funnel 2 RF	350.0 Vpp
Multipole RF	400.0 Vpp
Collision Cell	
Collision Energy	10.0 eV
Collision RF	2000.0 Vpp
Quadrupole	
Ion Energy	10.0 eV
Low Mass	*m/z* 450.00
Focus Pre TOF	
Transfer Time	100 µs
Pre Pulse Storage	10.0 µs

Table S2. Instrument Parameters for Negative and Positive Ion Mode LC-MS/MS Experiments.

**Negative Ion Mode**			**Positive Ion Mode**		
ESI			ESI		
Capillary	3600 V		Capillary	4500 V	
Nebulizer	2.0 Bar		Nebulizer	2.0 Bar	
Dry Gas	8.0 l/min		Dry Gas	8.0 l/min	
Dry Temp	220 °C		Dry Temp	220 °C	
MS/MS Stepping			MS/MS Stepping		
$1/K_0$	Scan 1	Scan 2	$1/K_0$	Scan 1	Scan 2
0.40	40 eV	40 eV	0.50	40 eV	40 eV
2.32	50 eV	65 eV	1.90	40 eV	40 eV
Collision RF	450 Vpp	1800 Vpp	Collision RF	500 Vpp	1500 Vpp
Transfer Time	25 µs	120 µs	Transfer Time	25 µs	85 µs
Pre Pulse Storage	5 µs	10 µs	Pre Pulse Storage	5 µs	8 µs

Table S3. Product Information for Antibodies.

Target	Company	Species	Clone	Lot	Cell Types/Structures	RRID	Catalog Number	Fluorophore
Collagen IV (α1/2)	Millipore Sigma	Mouse	7S Domain	3953676	Tubular Basement Membrane, Mesangial Matrix, and Glomerular Capsule	AB_2229703	MAB3326	None (uses secondary)
Collagen IV (α5)	Cosmo Bio USA	Rat	B51	002	Glomerular Basement Membrane, Glomerular Capsule, and Collecting Duct and Distal Convoluted Tubule Basement Membrane	AB_3065220	SGE-CFT451	FITC (did not work, used secondary)
Tensin	Sigma-Aldrich	Rabbit	TNS1	A115685	Mesangial Cells and Vascular Smooth Muscle Cells	AB_10796111	HPA036089	None (uses secondary)
Podocalyxin	Abcam	Rabbit	PODXL	1000363-1	Podocyte Cytoplasm and Plasma Membrane	AB_3065225	AB208254	AF 488
CD31	Abcam	Mouse	JC/7OA	1038052-1	Endothelial Cells	AB_2890260	AB215912	AF 647
Fibronectin	Abcam	Rabbit	Fibronectin	1054792-1	Mesangial Matrix and Muscularized Vessel Walls	AB_3086599	AB309604	AF 594
Synaptopodin	Abcam	Rabbit	Synaptopodin	GR3400313-3	Podocyte Cytoplasm and Plasma Membrane	AB_3086600	AB282117	Cy7 (conjugated with Lightning Link)
Nestin	Novus	Mouse	10C2	D127147	Podocyte Cytoplasm	AB_922042	NB300-266AF488	AF 488
αSMA	Abcam	Mouse	1A4	GR3395316-5	Vascular Smooth Muscle Cells	AB_2924381	AB202368	AF 594
AQP1	Abcam	Rabbit	Aquaporin 1	1028514-1	Proximal Tubules	AB_3086601	AB225225	AF 647
Secondary anti-rat	Jackson Immuno Research	Donkey	X	161323	X	AB_2340667	712-165-153	Cy3
Secondary anti-mouse	Jackson Immuno Research	Donkey	X	160571	X	AB_2340819	715-175-150	Cy5
Secondary anti-rabbit	Jackson Immuno Research	Donkey	X	162188	X	AB_2313584	711-545-152	AF488

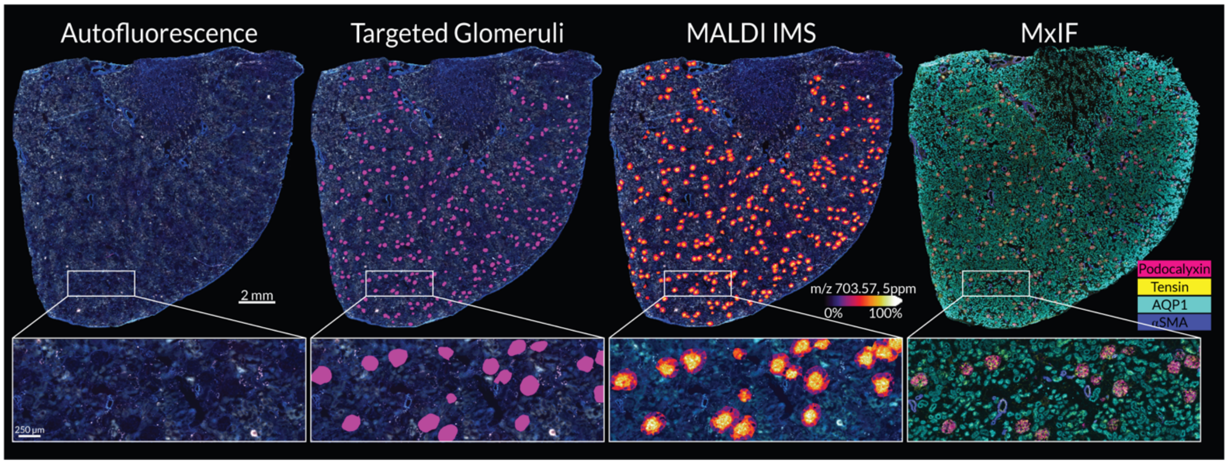

Figure S1. Overall imaging workflow. The whole-slide autofluorescence image of the tissue section (**A**) is used to segment glomeruli across the section (**B**). A targeted MALDI IMS experiment is performed on only glomerular regions (**C**). After the MALDI IMS experiment, the matrix can be removed and MxIF can be stained on the same tissue section.

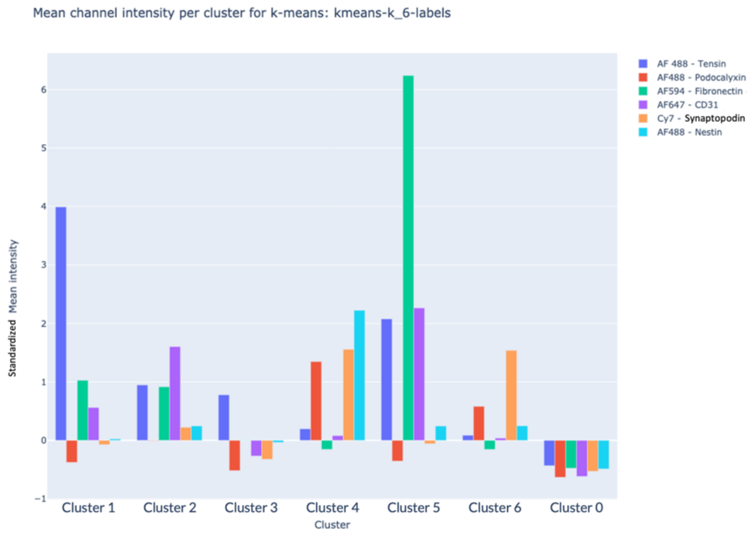

Figure S2. Plot of standardized mean fluorescent intensities of antibodies for each *k*-means cluster. Note, a non-cell specific cluster (cluster 3) includes tissue regions with low-level signal from tensin and minimal MxIF signal detected in other channels. These regions include pixels with seemingly high background signal and low cell type specificity. A zero/low MxIF intensity cluster (cluster 0) captures regions with minimal MxIF signal for this whole antibody marker panel. These low MxIF-signal regions may include tissue features not stained using the included antibodies.

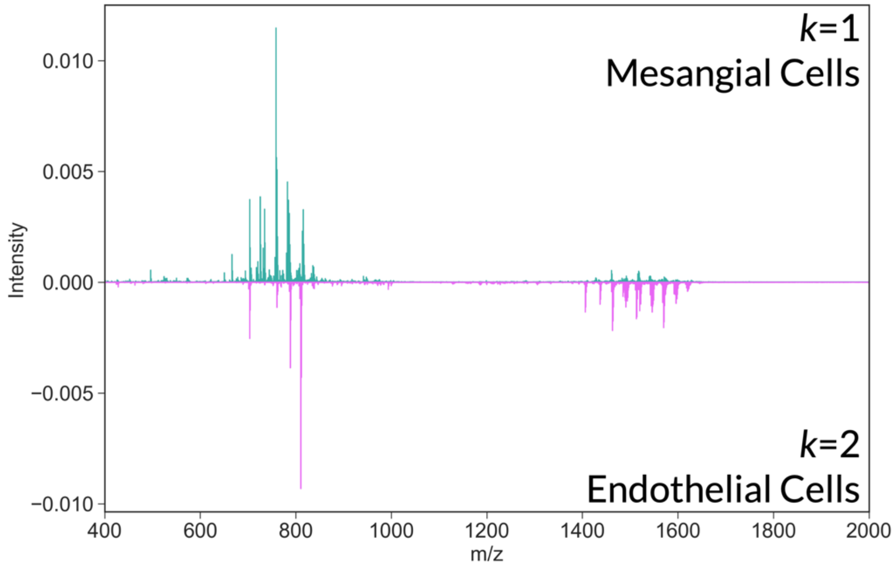

Figure S3. Difference spectrum showing average intensity differences between clusters 1 (mesangial cell-related segment) and 2 (endothelial cell-related segment).

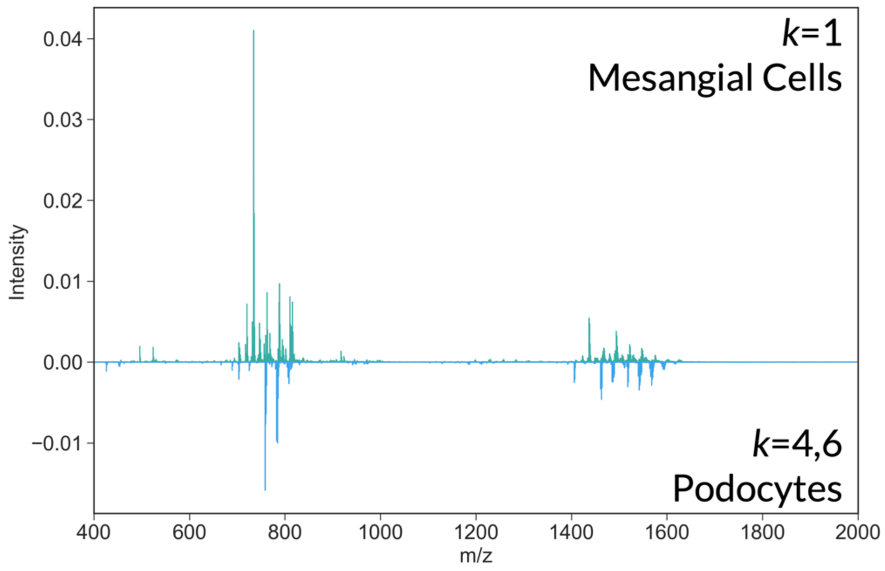

Figure S4. Difference spectrum showing average intensity differences between clusters 1 (mesangial cell-related segment) and a combined 4 and 6 (podocyte-related segment).

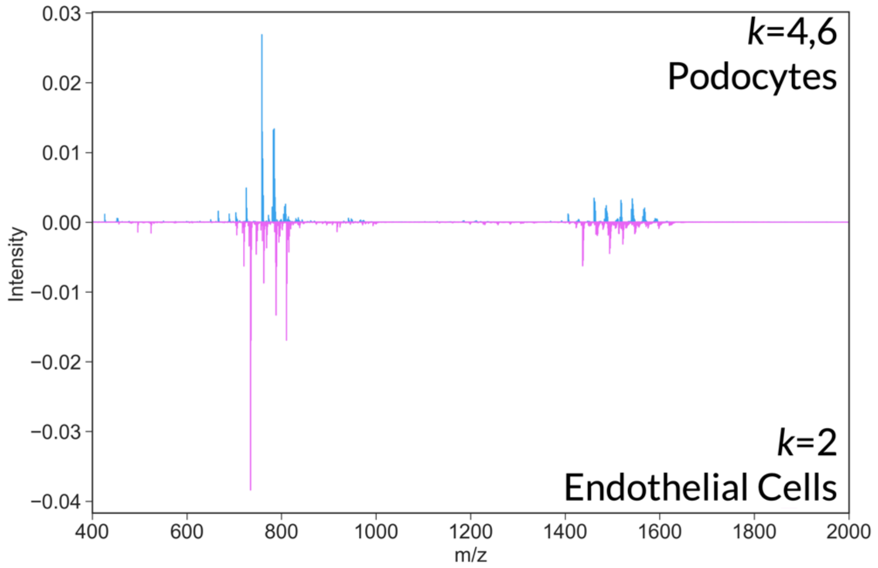

Figure S5. Difference spectrum showing average intensity differences between combined clusters 4 and 6 (podocyte-related segment) and 2 (endothelial cell-related segment).

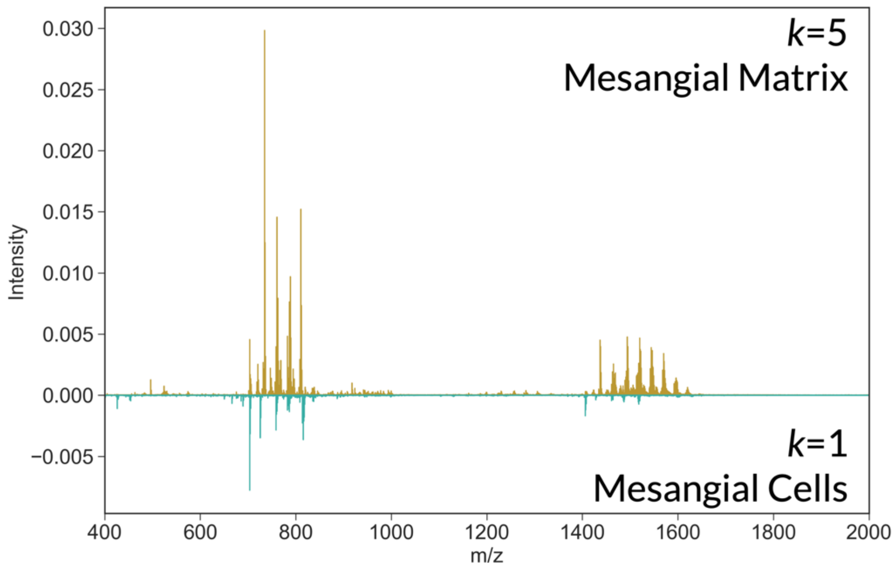

Figure S6. Difference spectrum showing average intensity differences between clusters 5 (mesangial matrix-related segment) and 1 (mesangial cell-related segment).

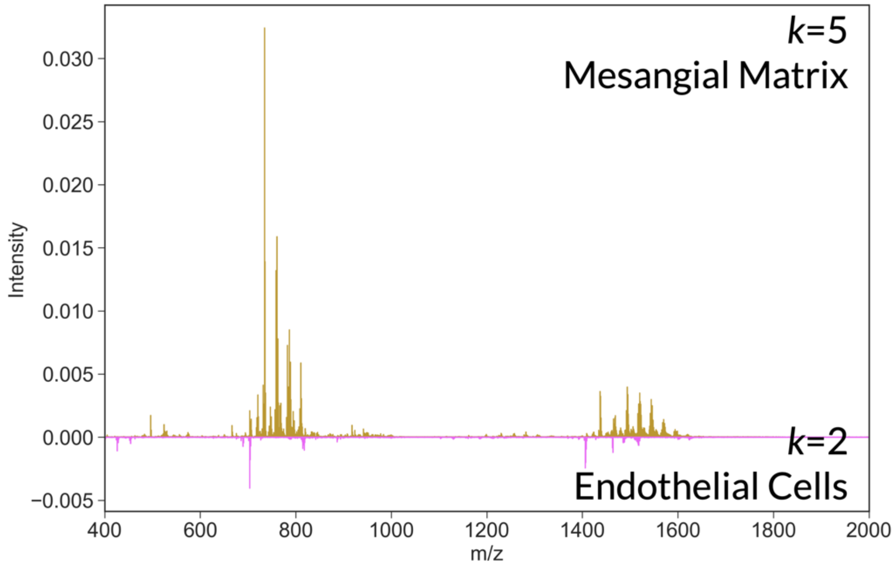

Figure S7. Difference spectrum showing average intensity differences between clusters 5 (mesangial matrix-related segment) and 2 (endothelial cell-related segment).

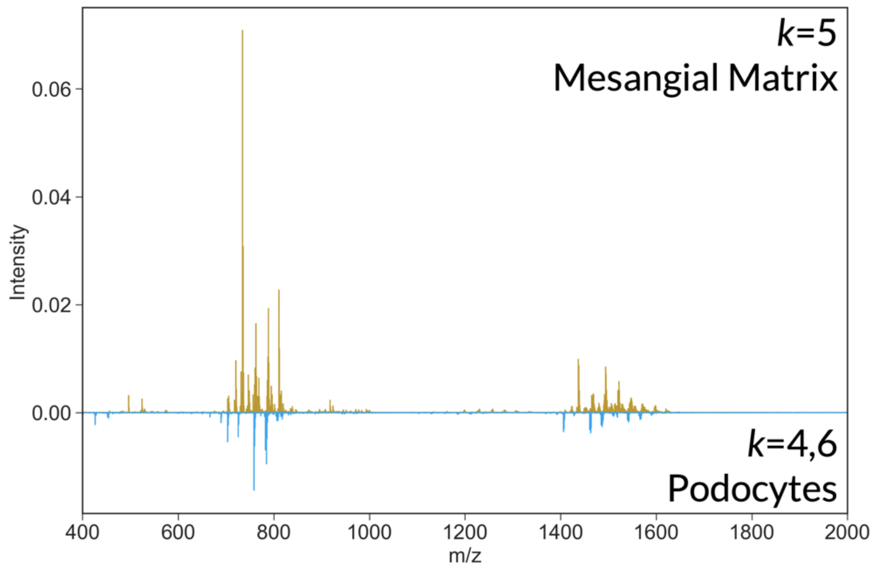

Figure S8. Difference spectrum showing average intensity differences between clusters 5 (mesangial matrix-related segment) and combined clusters 4 and 6 (podocyte-related segment).

Table S4. XGBoost Classification Model Performance Metrics per Cluster.

Cluster 1 - Mesangial Cells	Minimum	Maximum	Mean
Balanced Accuracy	0.977	0.9783	0.9777
F1-Score	0.9754	0.9767	0.976
Precision	0.9973	0.9975	0.9974
Recall	0.9542	0.9568	0.9556
Cluster 2 - Endothelial Cells			
Balanced Accuracy	0.9037	0.9051	0.9042
F1-Score	0.8812	0.8833	0.8821
Precision	0.9607	0.9635	0.9625
Recall	0.813	0.816	0.8142
Cluster 4 & 6 - Podocytes			
Balanced Accuracy	0.8872	0.888	0.8876
F1-Score	0.8812	0.8822	0.8817
Precision	0.8802	0.8812	0.8806
Recall	0.8812	0.8841	0.8827
Cluster 5 - Mesangial Matrix			
Balanced Accuracy	0.9997	0.9999	0.9998
F1-Score	0.987	0.9996	0.9991
Precision	0.998	0.9993	0.9986
Recall	0.9994	0.9999	0.9996

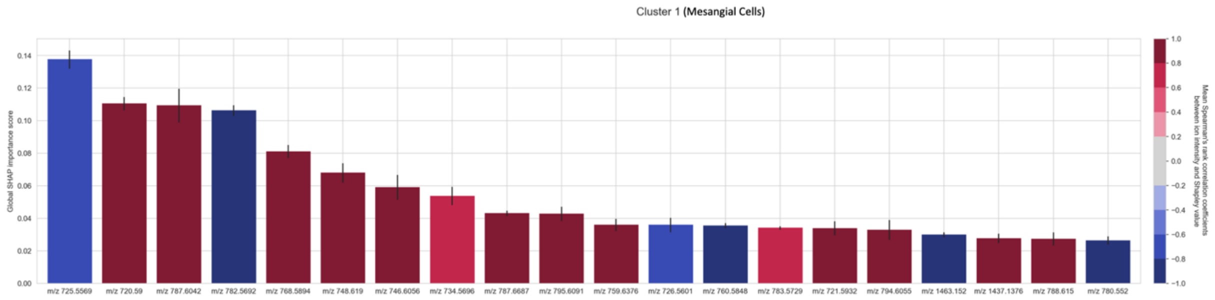

Figure S9. Global SHAP scores in ranked magnitude order for cluster 1 (primarily mesangial cells). The color of each bar reports whether that ion species tends to be positively (red) or negatively (blue) correlated to cluster 1.

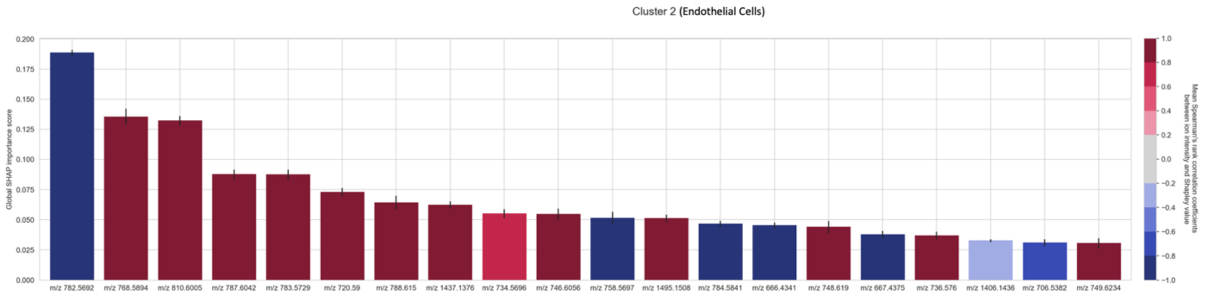

Figure S10. Global SHAP scores in ranked magnitude order for cluster 2 (primarily endothelial cells). The color of each bar reports whether that ion species tends to be positively (red) or negatively (blue) correlated to cluster 2.

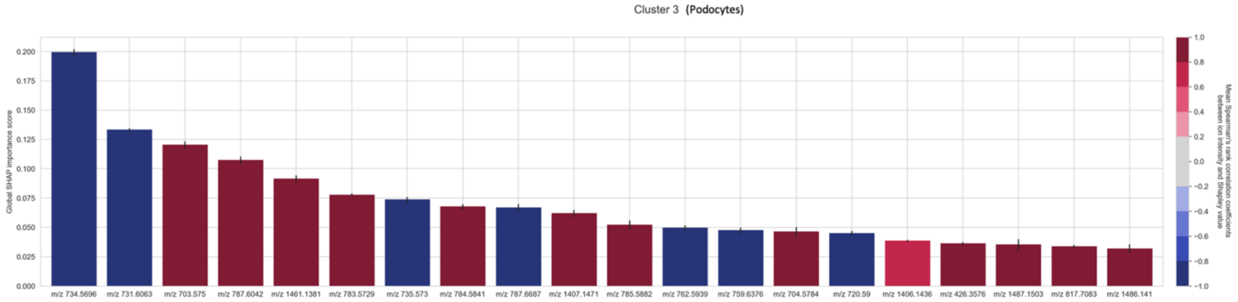

Figure S11. Global SHAP scores in ranked magnitude order for the combined clusters 4 and 6 (primarily podocytes). The color of each bar reports whether that ion species tends to be positively (red) or negatively (blue) correlated to clusters 4 and 6.

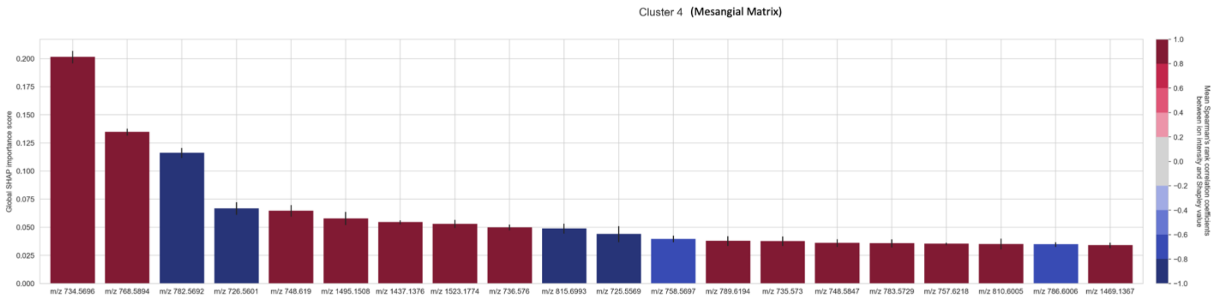

Figure S12. Global SHAP scores in ranked magnitude order for cluster 5 (primarily mesangial matrix). The color of each bar reports whether that ion species tends to be positively (red) or negatively (blue) correlated to cluster 5.

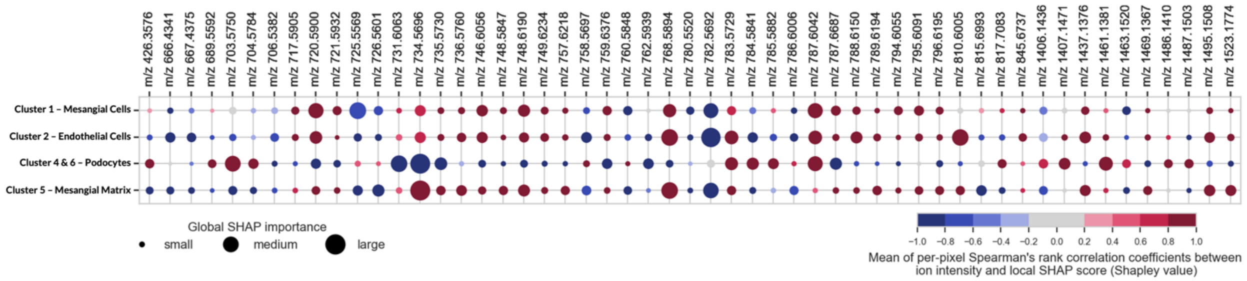

Figure S13. Full SHAP bubble plot, with m/z values and including isotopes and lipid dimers. The bubble plot summarizes the biomarker candidates for the glomerular segmentations and their dominant cell types. The size of each bubble indicates the global SHAP importance of a given ion species (column) to recognizing a given glomerular subarea (and its dominant cell type) (row), and the color indicates a positive (red) or negative (blue) correlation of the ion species abundance to that cluster's recognition. From this analysis, it is shown that every glomerular segmentation has its own unique profile of relevant ion species.

Table S5. Identifications of SHAP-analyzed *m/z* values

MALDI m/z	LC-MS/MS m/z	Theoretical m/z	MALDI ppm Error	LC-MS/MS ppm Error	Identification	Adduct Type
426.3576	426.3568	426.3578	-0.5	-2.3	CAR (18:1)	$[M+H]^+$
666.4341	666.4305	666.4341	0.0	-5.4	PS (27:0)	$[M+H]^+$
667.4375	x	x	x	x	Isotope	x
689.5592	689.5605	689.5592	0.0	1.9	CerPE (d36:1)	$[M+H]^+$
703.575	703.5757	703.5748	0.3	1.3	SM (d34:1)	$[M+H]^+$
704.5784	x	x	x	x	Isotope	x
706.5382	706.5381	706.5381	0.1	0.0	PC (30:0)	$[M+H]^+$
717.5905	x	717.5905	0.0	x	*CerPE (38:1)	$[M+H]^+$
720.59	x	720.5902	-0.3	x	*PC (O-32:0)	$[M+H]^+$
721.5932	x	x	x	x	Isotope	x
725.5569	x	725.556796	0.1	x	*SM (d34:1)	$[M+Na]^+$
726.5601	x	x	x	x	Isotope	x
731.6063	731.6072	731.6061	0.3	1.5	SM (d36:1)	$[M+H]^+$
734.5696	734.5672	734.5694	0.3	-3.0	PC (32:0)	$[M+H]^+$
735.573	x	x	x	x	Isotope	x
736.576	x	x	x	x	Isotope	x
746.6056	746.603	746.6058	-0.3	-3.8	PC (O-34:1)	$[M+H]^+$
748.5847	748.5851	748.5851	-0.5	0.0	PC (33:0)	$[M+H]^+$
748.619	748.6198	748.6215	-3.3	-2.3	PC (O-34:0)	$[M+H]^+$
749.6234	x	x	x	x	Isotope	x
757.6218	757.623	757.6218	0.0	1.6	SM (d38:2)	$[M+H]^+$
758.5697	758.5699	758.5694	0.4	0.7	PC (34:2)	$[M+H]^+$
759.6376	759.6359	759.6374	0.3	-2.0	SM (d38:1)	$[M+H]^+$
760.5848	760.5842	760.5851	-0.4	-1.2	PC (34:1)	$[M+H]^+$
762.5939	x	x	x	x	Isotope	x
768.5894	x	768.5902	-1.0	x	*PC (O-36:4)	$[M+H]^+$
780.552	780.5558	780.5538	-2.3	2.6	PC (36:5)	$[M+H]^+$
782.5692	782.5687	782.5694	-0.3	-0.9	PC (36:4)	$[M+H]^+$
783.5729	x	x	x	x	Isotope	x
784.5841	784.5872	784.5851	-1.3	2.7	PC (36:3)	$[M+H]^+$
785.5882	x	x	x	x	Isotope	x
786.6006	786.5972	786.6007	-0.1	-4.4	PC (36:2)	$[M+H]^+$
787.6042	x	x	x	x	Isotope	x
787.6687	787.6688	787.6687	0.0	0.1	SM (d40:1)	$[M+H]^+$
788.615	788.618	788.6164	-1.8	2.0	PC (36:1)	$[M+H]^+$
789.6194	x	x	x	x	Isotope	x
794.6055	x	794.6058	-0.4	x	*PC (O-38:5)	$[M+H]^+$
795.6091	x	x	x	x	Isotope	x
796.6195	796.6172	796.6215	-2.5	-5.4	PC (O-38:4)	$[M+H]^+$
810.6005	x	810.6007	-0.2	x	*PC (38:4)	$[M+H]^+$
815.6993	815.6976	815.7001	-1.0	-3.1	SM (d42:1)	$[M+H]^+$
817.7083	x	x	x	x	Unknown	x
845.6737	x	x	x	x	Unknown	x
1406.1436	x	x	x	x	Lipid Dimer	x
1407.1471	x	x	x	x	Lipid Dimer	x
1437.1376	x	x	x	x	Lipid Dimer	x
1461.1381	x	x	x	x	Lipid Dimer	x
1463.152	x	x	x	x	Lipid Dimer	x
1469.1367	x	x	x	x	Lipid Dimer	x
1486.141	x	x	x	x	Lipid Dimer	x
1487.1503	x	x	x	x	Lipid Dimer	x
1495.1508	x	x	x	x	Lipid Dimer	x
1523.1774	x	x	x	x	Lipid Dimer	x

Identified with exact mass from MALDI *m/z

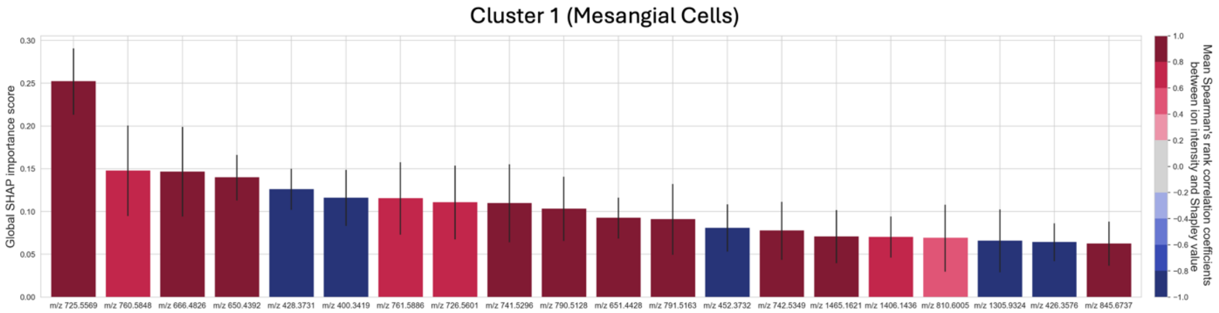

Figure S14. Global SHAP scores from a binary classification model of cluster 1 (primarily mesangial cells) comparing superficial and deep glomeruli in ranked magnitude order. The color of each bar reports whether that ion species is correlated with superficial glomeruli (red) or deep glomeruli (blue) in cluster 1. The tentative identification based on accurate mass for the top 5 ions is SM (d34:1) (*m/z* 725.56), PC (34:1) (*m/z* 760.58), unknown (*m/z* 666.48), unknown (*m/z* 650.44), and CAR (18:0) (*m/z* 428.37).

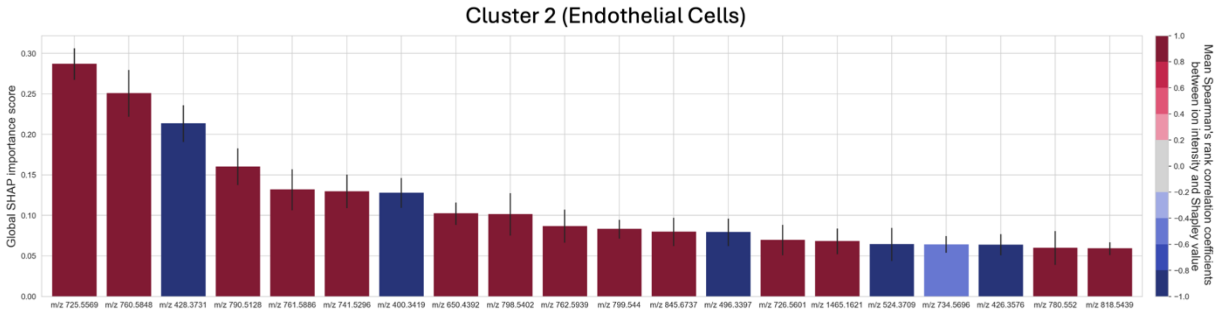

Figure S15. Global SHAP scores from a binary classification model of cluster 2 (primarily endothelial cells) comparing superficial and deep glomeruli in ranked magnitude order. The color of each bar reports whether that ion species is correlated with superficial glomeruli (red) or deep glomeruli (blue) in cluster 2. The tentative identification based on accurate mass for the top 5 ions is SM d34:1 (*m/z* 725.56), PC 34:1 (*m/z* 760.58), CAR 18:0 (*m/z* 428.37), unknown (*m/z* 790.51), and unknown (*m/z* 761.59).

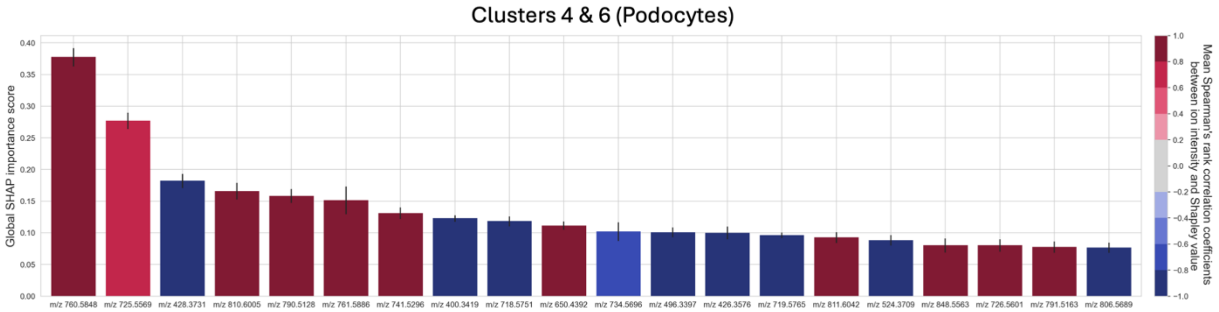

Figure S16. Global SHAP scores from a binary classification model of clusters 4 & 6 (primarily podocytes) comparing superficial and deep glomeruli in ranked magnitude order. The color of each bar reports whether that ion species is correlated with superficial glomeruli (red) or deep glomeruli (blue) in clusters 4 & 6. The tentative identification based on accurate mass for the top 5 ions is PC (34:1) (*m/z* 760.58), SM (d34:1) (*m/z* 725.56), CAR (18:0) (*m/z* 428.37), PC (38:4) (*m/z* 810.60), and unknown (*m/z* 790.51).

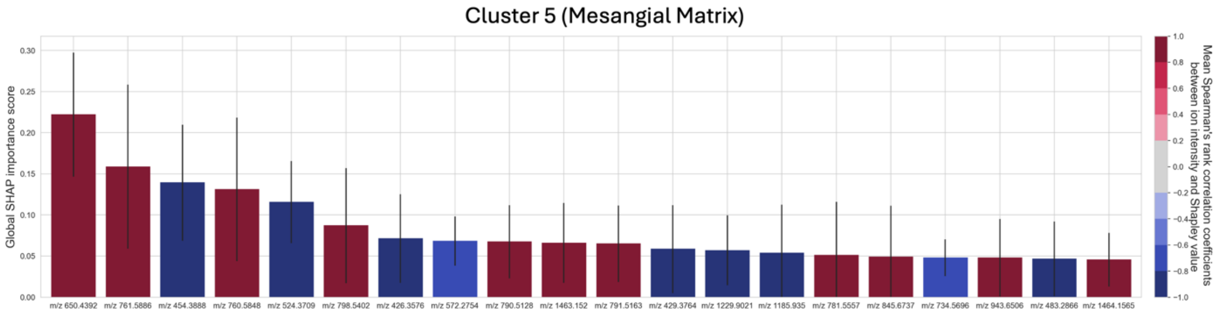

Figure S17. Global SHAP scores from a binary classification model of cluster 5 (primarily mesangial matrix) comparing superficial and deep glomeruli in ranked magnitude order. The color of each bar reports whether that ion species is correlated with superficial glomeruli (red) or deep glomeruli (blue) in cluster 5. The tentative identification based on accurate mass for the top 5 ions is unknown (*m/z* 650.44), unknown (*m/z* 761.59), CAR (20:1) (*m/z* 454.39), PC (34:1) (*m/z* 760.58), and LPC (18:0) (*m/z* 524.37).

Table S6. XGBoost Binary Classification Model Performance Metrics per Cluster.

Cluster 1 - Mesangial Cells	Minumum	Maximum	Mean
Balanced Accuracy	0.8805	0.892	0.8862
F1-Score	0.8266	0.8414	0.8344
Precision	0.7567	0.78	0.768
Recall	0.9057	0.9218	0.9135
Cluster 2 - Endothelial Cells			
Balanced Accuracy	0.9218	0.9277	0.924
F1-Score	0.926	0.9311	0.9279
Precision	0.9258	0.9368	0.9308
Recall	0.9207	0.9307	0.925
Clusters 4 & 6 - Podocytes			
Balanced Accuracy	0.9267	0.9293	0.9279
F1-Score	0.9249	0.928	0.9264
Precision	0.9359	0.944	0.9395
Recall	0.9092	0.9178	0.9138
Cluster 5 - Mesangial Matrix			
Balanced Accuracy	0.8309	0.8547	0.8439
F1-Score	0.7481	0.7779	0.7634
Precision	0.6317	0.6828	0.6634
Recall	0.877	0.9332	0.8997

References

1. Neumann E, Allen J, Brewer M, et al. VU Biomolecular Multimodal Imaging Center (BIOMIC) kidney characterization pipeline for tissues collected through the Cooperative Human Tissue Network (CHTN). Published 2020. Accessed January 29, 2024. https://www.protocols.io/view/vu-biomolecular-multimodal-imaging-center-biomic-k-dm6gpreo8vzp/v4
2. Anderson D, Dufresne M, Messinger JD, et al. Matrix Sublimation via In-House Developed Sublimation Apparatus. Published April 5, 2023. Accessed April 5, 2023. https://www.protocols.io/view/matrix-sublimation-via-in-house-developed-sublimat-n92ldpyn9l5b/v1
3. Patterson NH, Tuck M, Lewis A, et al. Next Generation Histology-Directed Imaging Mass Spectrometry Driven by Autofluorescence Microscopy. *Anal Chem*. 2018;90:12404-12413. doi:10.1021/acs.analchem.8b02885
4. Esselman AB, Patterson NH, Migas LG, et al. Microscopy-Directed Imaging Mass Spectrometry for Rapid High Spatial Resolution Molecular Imaging of Glomeruli. *J Am Soc Mass Spectrom*. 2023;34(7):1305-1314. doi:10.1021/JASMS.3C00033/SUPPL_FILE/JS3C00033_SI_001.ZIP
5. Patterson NH, Neumann EK, Sharman K, et al. Autofluorescence microscopy as a label-free tool for renal histology and glomerular segmentation. *bioRxiv*. Published online 2021. doi:10.1101/2021.07.16.452703
6. Spraggins JM, Djambazova K V, Rivera ES, et al. High-Performance Molecular Imaging with MALDI Trapped Ion-Mobility Time-of-Flight (timsTOF) Mass Spectrometry. *Anal Chem*. 2019;91:14552. doi:10.1021/acs.analchem.9b03612
7. Brewer M, McDonough L, Zhu Y, et al. Multiplex Cyclical Immunofluorescence on Fresh Frozen Tissue-V3. Published 2023. Accessed December 17, 2023. https://www.protocols.io/view/multiplex-cyclical-immunofluorescence-on-fresh-fro-81wgb1m3yvpk/v3
8. Neumann E, Allen J, Harvey J, et al. PAS Staining of Fresh Frozen or Paraffin Embedded Human Kidney Tissue. protocols.io. doi:10.17504/PROTOCOLS.IO.BUAKNSCW
9. Patterson H, Manz T. NHPatterson/wsireg: wsireg v0.3.0. Zenodo. doi:10.5281/ZENODO.5518238
